# Is the current ASAS expert definition of a positive family history useful in identifying axial spondyloarthritis? Results from the SPACE and DESIR cohorts

**DOI:** 10.1186/s13075-017-1335-8

**Published:** 2017-05-31

**Authors:** Zineb Ez-Zaitouni, Andrea Hilkens, Laure Gossec, Inger Jorid Berg, Robert Landewé, Roberta Ramonda, Maxime Dougados, Désirée van der Heijde, Floris van Gaalen

**Affiliations:** 1 Department of Rheumatology, Leiden University Medical Center, P.O. Box 9600, 2300 RC Leiden, The Netherlands; 2 Sorbonne Universités, UPMC Univ Paris 06, GRC-08, Paris, France; 3 Rheumatology Department, Pitie-Salpétrière Hospital, AP-HP, Paris, France; 4 Department of Rheumatology, Diakonhjemmet Hospital, Oslo, Norway; 5 Department of Clinical Immunology and Rheumatology, Amsterdam Medical Center, Amsterdam, The Netherlands; 6 Rheumatology Unit, Department of Medicine DIMED, University of Padova, Padova, Italy; 7 Paris Descartes University, Hôpital Cochin, Assistance Publique-Hôpitaux de Paris, INSERM (U1153): Epidémiologie Clinique et Biostatistiques, PRES Sorbonne Paris-Cité, Paris, France

**Keywords:** Family history, Ankylosing spondylitis, Acute anterior uveitis, Reactive arthritis, IBD, Psoriasis, Axial spondyloarthritis, Diagnostic work-up, Chronic back pain

## Abstract

**Background:**

The Assessment of SpondyloArthritis international Society (ASAS) definition of a positive family history (PFH) of spondyloarthritis (SpA) includes the following diseases in first- or second-degree relatives: ankylosing spondylitis (AS), acute anterior uveitis (AAU), reactive arthritis (ReA), inflammatory bowel disease (IBD), and psoriasis. However, it is not known if a PFH for each of these diseases contributes to making a diagnosis of axSpA, sacroiliitis on imaging, or fulfilling the ASAS criteria in patients presenting with chronic back pain (CBP). Therefore, the aim of this study was to assess which SpA diseases in family members are associated with human leukocyte antigen B27 (HLA-B27) and axial spondyloarthritis (axSpA) in CBP patients.

**Methods:**

CBP patients suspected of axSpA from the SPACE (*n* = 438) and the DESIR (*n* = 647) cohort were asked about the presence of SpA diseases in first- or second-degree relatives (AS, AAU, ReA, IBD, and psoriasis). The associations between a PFH and HLA-B27, sacroiliitis on imaging (magnetic resonance imaging (MRI) or radiographs), axSpA diagnosis, and ASAS classification in CBP patients were assessed.

**Results:**

In the SPACE and the DESIR cohort, a PFH of AS (odds ratio (OR) 5.9 (95% confidence interval (CI) 3.5–9.9), and OR 3.3 (95% CI 2.1–5.2)) and a PFH of AAU (OR 9.8 (95% CI 3.3–28.9) and OR 21.6 (95% CI 2.9–160.1)) were significantly associated with presence of HLA-B27. Furthermore, in both cohorts a PFH of AS and a PFH of AAU were positively associated with fulfilment of the ASAS criteria, but not with sacroiliitis on imaging. In SPACE but not in DESIR a PFH of AAU was positively associated with axSpA diagnosis. In both cohorts a PFH of ReA, IBD, or psoriasis was not positively associated with HLA-B27 positivity, sacroiliitis on imaging, axSpA diagnosis, or meeting the ASAS criteria for axSpA.

**Conclusions:**

In our cohorts, a PFH of AS or AAU is useful for case-finding of axSpA as this is correlated with HLA-B27 carriership. However, as a PFH of ReA, IBD, or psoriasis does not contribute to identifying axSpA in CBP patients, these data suggest that the widely used ASAS definition of a PFH of SpA should be updated.

**Trial registration:**

Trial registration number, NCT01648907. Registered on 20 July 2012.

**Electronic supplementary material:**

The online version of this article (doi:10.1186/s13075-017-1335-8) contains supplementary material, which is available to authorized users.

## Background

Axial spondyloarthritis (axSpA) is a chronic inflammatory disease mainly involving the spine and sacroiliac joints. Genetic risk factors play a role in axSpA with human leukocyte antigen B27 (HLA-B27) by far the strongest genetic risk factor for disease [1]. A positive family history (PFH) of SpA has been reported in up to 40% of ankylosing spondylitis (AS) patients and the risk to develop AS in HLA-B27-positive first-degree relatives of HLA-B27-positive AS patients has been estimated to be 16-times higher than that of HLA-B27-positive individuals in the general population [2–5]. As a result, familial aggregation of SpA is considered a risk indicator for the presence of axSpA in patients with chronic back pain (CBP) and is part of several SpA classification criteria sets [6–8].

In the Assessment of SpondyloArthritis international Society (ASAS) classification criteria for axSpA, a PFH of SpA is defined as the presence of any of the following diseases in first- or second-degree relatives: AS, acute anterior uveitis (AAU), reactive arthritis (ReA), inflammatory bowel disease (IBD), and psoriasis [8]. This PFH definition is also recommended in diagnosing axSpA, and is incorporated in several referral strategies for CBP patients used by nonrheumatologists [9, 10].

However, only three of these diseases have a documented HLA-B27 association (i.e., AS, AAU, and ReA) and two are not HLA-B27 associated (i.e., IBD and psoriasis) [11–13]. Thus, even though a PFH of SpA is a common finding in axSpA patients it is unknown whether a PFH of each of these diseases contributes equally well to making a diagnosis of axSpA in patients presenting with CBP.

In this study, we present data from two unique early axSpA cohorts: the multinational multicenter SPondyloArthritis Caught Early (SPACE) cohort and the French multicenter DEvenir des Spondylarthropathies Indifférenciées Récentes (DESIR) cohort. In these cohorts of CBP patients with a suspicion of axSpA we have investigated which of the SpA diseases present among family members were associated with HLA-B27, sacroiliitis on imaging, a clinical diagnosis of axSpA, and meeting the ASAS classification criteria for axSpA.

## Methods

### Patient cohorts

The SPACE cohort is a prospective study which includes patients with short-term CBP (≥3 months, ≤2 years, and an onset <45 years) at a minimum age of 16 years from five Rheumatology outpatient clinics in the Netherlands, Norway, and Italy. DESIR is a prospective longitudinal cohort running in 25 centers in France (clinicaltrials.gov, NCT01648907). Patients between the ages of 18 and 50 years with inflammatory back pain (IBP) according to the Calin [14] or Berlin [15] criteria, persisting ≥3 months but <3 years, were included. In addition, the treating rheumatologist had to have a substantial suspicion of axSpA (level of confidence ≥5 on a 0–10 rating scale, where 0 = not confident and 10 = very confident). A detailed description of both cohorts is provided elsewhere [16, 17].

Both studies were approved by local medical ethics committees. All participants provided prior written informed consent.

### Clinical data collection

All patients underwent a diagnostic work-up according to a fixed protocol which includes physical examination, laboratory assessments (HLA-B27, C-reactive protein (CRP), and erythrocyte sedimentation rate (ESR)), and magnetic resonance imaging (MRI) as well as radiographs of sacroiliac joints. The presence and history of clinical SpA features were assessed: IBP, good response to nonsteroidal anti-inflammatory drugs (NSAIDs), peripheral arthritis, dactylitis, enthesitis, AAU, IBD, and psoriasis. Patients were asked about the presence of any of the following SpA diseases in first- or second-degree relatives: AS, AAU, ReA, IBD, and psoriasis. For each SpA disease the possible answers were “yes”, “no” or “unknown/uncertain”. A PFH was defined as the presence of ≥1 SpA-related disease in first- (mother, father, sister, brother, daughter, son) or second-degree relatives (aunt, uncle, niece, nephew, grandmother, and grandfather) reported by the patient [8].

In the SPACE cohort, axSpA was diagnosed by the treating rheumatologist. In the DESIR cohort axSpA diagnosis was defined as the level of confidence regarding diagnosis of ≥8 on a 0–10 numerical rating scale (where 0 = not confident at all and 10 = very confident). The ASAS axSpA classification criteria were used to classify patients.

### Data analysis

Baseline demographic and clinical characteristics are presented using descriptive statistics for both the SPACE and DESIR cohorts. The association between each PFH disease and HLA-B27 in patients was assessed using the Chi-squared test. Similar analyses were performed for the assessment of the association between each PFH disease and a clinical diagnosis of axSpA, sacroiliitis on imaging (defined as either sacroiliitis on MRI, radiographs, or on both modalities by local reading), and the fulfilment of the ASAS axSpA classification criteria.

Statistical testing was performed using Stata SE v.14 (StataCorp LP, College Station, TX, USA).

## Results

For the current analyses, 438 patients from the SPACE cohort and 647 patients from the DESIR cohort with complete data at baseline were used (Table 1). Several baseline characteristics differed between the two cohorts, mainly reflecting differences in inclusion criteria. In both cohorts, the distribution of all PFH diseases (AS, AAU, ReA, IBD, and psoriasis) in first- or second-degree relatives were similar.

**Table 1:** Baseline characteristics and clinical features of CBP patients in the SPACE (*n* = 438) and DESIR cohorts (*n* = 647)

Characteristic	SPACE *n* = 438	DESIR *n* = 647
Age, years	31.3 (8.3)	33.6 (8.6)
Symptom duration, months	13.4 (7.4)	18.1 (10.5)
Male	165 (38)	305 (47)
IBP	286 (66)	647 (100)*
Good response to NSAIDs^a^	181/420 (43)	515/643 (80)
Past history or current symptoms		
Peripheral arthritis	64 (15)	363 (56)
Dactylitis	24 (6)	83 (13)
Enthesitis	89 (20)	312 (48)
AAU	34 (8)	51 (8)
IBD	34 (8)	23 (4)
Psoriasis	51 (12)	97 (15)
Elevated CRP (mg/L)/ESR (mm)^b^	123 (28)	254 (39)
HLA-B27 positive	174/436 (40)	376/646 (58)
Sacroiliitis, radiography^c^	48/434 (11)	172 (27)
Sacroiliitis, MRI^c^	135/431 (31)	207/636 (33)
ASAS criteria for axSpA	203 (46)	410/634 (65)
Any positive family history^d^	185 (42)	249 (39)
PFH of AS	90 (21)	127 (20)
PFH of AAU	27 (6)	29 (5)
PFH of ReA	14 (3)	6 (1)
PFH of IBD	33 (8)	32 (5)
PFH of Psoriasis	83 (19)	129 (20)
Total number of SpA diseases in first- or second-degree relatives		
Number of patients with 1 disease	135 (73)	189 (76)
Number of patients with 2 diseases	39 (21)	47 (19)
Number of patients with ≥3 diseases	11 (6)	13 (5)

Unless specified otherwise, results are presented as mean ± SD or number (%)

*Inclusion criterion

^a^Back pain not present anymore or is much better 24–48 hours after a full dose of NSAID

^b^Values greater than the upper limit of normal

^c^Imaging based on local reading. of sacroiliac joints

^d^Presence in first- or second-degree relatives of any of the following: AS, AAU, ReA, IBD, or psoriasis

*AAU* acute anterior uveitis, *AS* ankylosing spondylitis, *ASAS axSpA* criteria, Assessment of Spondyloarthritis international Society criteria for axial Spondyloarthritis, *CBP* chronic back pain, *CRP* C-reactive protein, *ESR* erythrocyte sedimentation rate, *HLA-B27* human leucocyte antigen B27, *IBD* inflammatory bowel disease, *IBP* inflammatory back pain, *MRI* magnetic resonance imaging, *NSAID* non-steroidal anti-inflammatory drug, *PFH* positive family history, *ReA* reactive arthritis, *SpA* spondyloarthritis

In both the SPACE and DESIR cohort, any PFH, a PFH of AS, and a PFH of AAU were significantly associated with HLA-B27 in CBP patients (Table 2). In multivariable analyses, a PFH of AS or AAU were independently associated with HLA-B27 positivity (data not shown). However, in neither cohort was a PFH of ReA, IBD, or psoriasis associated with HLA-B27-positivity.

**Table 2:** Association of family history manifestations with HLA-B27 in CBP patients in the SPACE (*n* = 438) and DESIR cohorts (*n* = 647)

	SPACE*				DESIR**			
	HLA-B27 + *n* = 174	HLA-B27– *n* = 262	OR (95% CI)	*P* value	HLA-B27 + *n* = 376	HLA-B27– *n* = 270	OR (95% CI)	*P* value
Any PFH	97	87	2.5 (1.7–3.8)	<0.001	158	91	1.4 (1.0–2.0)	0.032
AS	65	24	5.9 (3.5–9.9)	<0.001	100	27	3.3 (2.1–5.2)	<0.001
AAU	23	4	9.8 (3.3–28.9)	<0.001	28	1	21.6 (2.9–160.1)	0.003
ReA	5	9	0.8 (0.3–2.5)	0.745	1	5	0.1 (0.01–1.2)	0.075
IBD	12	21	0.9 (0.4–1.8)	0.666	17	15	0.8 (0.4–1.6)	0.551
Psoriasis	34	48	1.1 (0.6–1.8)	0.750	69	60	0.8 (0.5–1.2)	0.225

*Two patients with unknown HLA-B27 status

**One with patient unknown HLA-B27 status

*AAU* acute anterior uveitis, *AS* ankylosing spondylitis, *CBP* chronic back pain, *CI* confidence interval, *HLA-B27* human leukocyte antigen B27, *IBD* inflammatory bowel disease, *OR* odds ratio, *PFH* positive family history (manifestation in first- or second-degree relatives), *ReA* reactive arthritis

To investigate whether the presence of a PFH for any of the diseases in relatives is associated with sacroiliitis, a clinical diagnosis of axSpA, or a positive ASAS classification, similar analyses were performed. In both cohorts, neither ‘any PFH’ nor a separate PFH of a disease were associated with sacroiliitis (Additional file 1). In SPACE but not in DESIR a PFH of AAU was positively associated with axSpA diagnosis (Additional file 2). While a PFH of AS or AAU had a significant positive association with fulfilment of the ASAS criteria, such an association was not found for a PFH of ReA, IBD, or psoriasis in the SPACE and DESIR cohorts (Table 3).

In addition, the relative contribution of HLA-B27 was investigated among patients who were classified according to the ASAS criteria. A total of 203 (46%) patients from the SPACE cohort and 410 (63%) patients from the DESIR cohort fulfilled the ASAS criteria for axSpA, and 156 (77%) and 347 (85%) patients were HLA-B27 positive, respectively (Table 4). A PFH was reported more frequently in HLA-B27-positive patients than in HLA-B27-negative patients meeting the ASAS classification criteria (SPACE 45% vs 7% and DESIR 35% vs 6%).

**Table 3:** Association of family history manifestations with the fulfilment of ASAS axSpA criteria in the SPACE cohort and DESIR cohorts

	Fulfilment of ASAS axSpA criteria			
	SPACE		DESIR	
	OR (95% CI)	*P*- value	OR (95% CI)	*P*- value
Any PFH	2.1 (1.4-3.1)	<0.001	1.3 (1.0-1.9)	0.091
AS	3.3 (2.0-5.3)	<0.001	2.1 (1.3-3.3)	<0.001
AAU	7.4 (2.5-21.7)	<0.001	5.0 (1.5-16.7)	0.009
ReA	0.6 (0.2-1.9)	0.421	0.3 (0.05-1.5)	0.132
IBD	1.1 (0.5-2.2)	0.798	0.8 (0.4-1.6)	0.521
Psoriasis	1.2 (0.8-2.0)	0.388	1.2 (0.8-1.8)	0.464

Any PFH, any family history manifestation in first- or second-degree relatives; *AS* ankylosing spondylitis, *AAU* acute anterior uveitis, *ReA* reactive arthritis, *IBD* inflammatory bowel disease, *OR* odds ratio, *95% CI* 95% confidence interval

**Table 4:** PFH of diseases in first- or second-degree relatives of CBP patients meeting the ASAS axSpA criteria, stratified according to HLA-B27 status

Positive family history	SPACE cohort *n* = 201*		DESIR cohort *n* = 409**	
	HLA-B27+	HLA-B27–	HLA-B27+	HLA-B27–
Any PFH	90 (45%)	14 (7%)	145 (35%)	24 (6%)
AS	59 (29%)	2 (1%)	92 (23%)	5 (1%)
AAU	22 (11%)	1 (1%)	26 (6%)	0 (0)
ReA	4 (1%)	1 (1%)	1 (0.2%)	1 (0.2%)
IBD	11 (6%)	5 (3%)	14 (3%)	5 (1%)
Psoriasis	32 (16%)	9 (5%)	65 (16%)	20 (5%)

*Two hundred and three patients fulfilled ASAS-criteria, two with unknown HLA-B27 status

**Four hundred and ten patients fulfilled ASAS-criteria, one with unknown HLA-B27 status

*AAU* acute anterior uveitis, *AS* ankylosing spondylitis, *ASAS axSpA* Assessment of Spondyloarthritis international Society criteria for axial spondyloarthritis, *HLA-B27* human leucocyte antigen B27, *IBD* inflammatory bowel disease, *PFH* postitive family history (manifestation in first- or second-degree relatives), *ReA* reactive arthritis

## Discussion

To our knowledge this is the first study to investigate the usefulness of the separate SpA diseases in a PFH as defined for the ASAS classification criteria. In two independent cohorts of predominantly Caucasoid Europeans, we found that in CBP patients suspected of axSpA a PFH of ReA, IBD, or psoriasis were neither associated with HLA-B27 positivity, nor with sacroiliitis, a diagnosis of axSpA, or fulfilment of the ASAS criteria. In contrast, a PFH of AS or AAU was strongly correlated with HLA-B27 carriership.

IBD and psoriasis are generally not HLA-B27-associated diseases, but ReA has been reported to be associated with HLA-B27 in a secondary care setting, although in population-based studies the prevalence of HLA-B27 in ReA was comparable to that of the general population [12, 18]. A possible explanation for the absence of the association between ReA and HLA-B27 in our study is that the (self)-reported prevalence of ReA in family members of patients in the SPACE and DESIR cohort was low, suggesting underreporting.

It is important to emphasize that the current study was performed in patients with predominantly axial symptoms. Although only a PFH of AS or AAU have been shown to be independently associated with HLA-B27, this does not mean that a PFH of the other SpA diseases is always irrelevant [19]. For example, the presence of psoriasis in relatives could be relevant in a patient with peripheral symptoms suspected of psoriatic arthritis [20].

A strength of this study is the use of two large early axSpA cohorts in which all patients were assessed following a similar protocol which allowed for replication of findings. Strikingly similar prevalences of PFH were found in both cohorts which adds to the credibility of the data. The major limitation of this study, however, is that the diagnosis in relatives (the PFH) is solely based on patients’ information which may have led to either under- or overestimation of a PFH. However, this is similar to most clinical settings in which PFH is also mostly based on self-reporting. Moreover, subdividing a PFH into five different diseases meant that a PFH of, for instance, AAU or ReA was uncommon.

## Conclusions

In conclusion, in our two cohorts a PFH of ReA, IBD, and psoriasis does not contribute to diagnosing axSpA in CBP patients suspected of axSpA. A PFH of AS or AAU may be useful in case-finding in low prevalence settings, such as general practice, as these are correlated with HLA-B27 carriership. When replicated, preferably in other regions of the world in patients with a different genetic background, it is justified to remove a PFH of ReA, IBD, and psoriasis from the current ASAS definition of a positive PFH relevant for axSpA.

## Additional files

Additional file 1: Table S1. Association of family history manifestations with any positive imaging (sacroiliitis on MRI or radiographs) in the SPACE cohort and DESIR cohorts. (DOCX 19 KB)

Additional file 2: Table S2. Association of family history manifestations with clinical diagnosis in the SPACE cohort and DESIR cohorts. (DOCX 19 kb)

## Abbreviations

AAU: Acute anterior uveitis
AS: Ankylosing spondylitis
ASAS: Assessment of SpondyloArthrtis international Society
axSpA: Axial spondyloarthritis
CBP: Chronic back pain
HLA-B27: Human leukocyte antigen B27
IBD: Inflammatory bowel disease
IBP: Inflammatory back pain
MRI: Magnetic resonance imaging
PFH: Positive family history
ReA: Reactive arthritis
