# Supplementary material for: Is the current ASAS expert definition of a positive family history useful in identifying axial spondyloarthritis? Results from the SPACE and DESIR cohorts
Source: Arthritis Res Ther. 2017 May 31;19:118. doi: 10.1186/s13075-017-1335-8 (PMC5452625; doi:10.1186/s13075-017-1335-8)
Supplement: Supplementary file 1 — Association of family history manifestations with any positive imaging (sacroiliitis on MRI or radiographs) in the SPACE cohort and DESIR cohorts. (DOCX 19 KB) [file 13075_2017_1335_MOESM1_ESM.docx]

**Supplementary Table 1** Association of family history manifestations with any positive imaging (sacroiliitis on MRI or radiographs) in the SPACE cohort and DESIR cohorts.

|  | **Sacroiliitis on imaging (local reading)** | | | |
| --- | --- | --- | --- | --- |
|  | **SPACE** | | **DESIR** | |
|  | OR (95% CI) | *P*- value | OR (95% CI) | *P*- value |
| Any PFH | 0.9 (0.6-1.3) | 0.597 | 0.9 (0.7-1.3) | 0.753 |
| AS | 0.8 (0.5-1.4) | 0.452 | 1.0 (0.7-1.5) | 0.928 |
| AAU | 1.7 (0.8-3.8) | 0.164 | 1.2 (0.6-2.6) | 0.566 |
| ReA | 0.2 (0.02-1.2) | 0.075 | 0.8 (0.1-4.2) | 0.749 |
| IBD | 0.8 (0.4-1.7) | 0.530 | 0.7 (0.3-1.5) | 0.318 |
| Psoriasis | 0.9 (0.6-1.6) | 0.851 | 0.9 (0.6-1.4) | 0.652 |

Any PFH, any family history manifestation in first- or second-degree relatives; AS, ankylosing spondylitis; AAU, acute anterior uveitis; ReA, reactive arthritis; IBD, inflammatory bowel disease; OR, odds ratio; 95% CI, 95% confidence interval.
