# Supplementary material for: Is the current ASAS expert definition of a positive family history useful in identifying axial spondyloarthritis? Results from the SPACE and DESIR cohorts
Source: Arthritis Res Ther. 2017 May 31;19:118. doi: 10.1186/s13075-017-1335-8 (PMC5452625; doi:10.1186/s13075-017-1335-8)
Supplement: Supplementary file 2 — Association of family history manifestations with clinical diagnosis in the SPACE cohort and DESIR cohorts. (DOCX 19 kb) [file 13075_2017_1335_MOESM2_ESM.docx]

**Supplementary Table 2** Association of family history manifestations with clinical diagnosis in the SPACE cohort and DESIR cohorts.

|  | **Clinical axSpA diagnosis** | | | |
| --- | --- | --- | --- | --- |
|  | **SPACE** | | **DESIR*** | |
|  | OR (95% CI) | *P*- value | OR (95% CI) | *P*- value |
| Any PFH | 1.5 (1.0-2.2) | 0.053 | 1.0 (0.7-1.4) | 0.900 |
| AS | 1.3 (0.8-2.1) | 0.282 | 1.2 (0.8-1.7) | 0.461 |
| AAU | 3.4 (1.3-8.6) | 0.010 | 1.4 (0.7-3.0) | 0.350 |
| ReA | 0.2 (0.1-0.9) | 0.035 | 1.1 (0.2-5.7) | 0.870 |
| IBD | 1.0 (0.5-2.1) | 0.960 | 1.0 (0.5-2.1) | 0.982 |
| Psoriasis | 1.6 (1.0-2.6) | 0.064 | 0.9 (0.6-1.3) | 0.526 |

*Diagnosis defined as level of confidence of axSpA diagnosis ≥8. Any PFH, any family history manifestation in first- or second-degree relatives; AS, ankylosing spondylitis; AAU, acute anterior uveitis; ReA, reactive arthritis; IBD, inflammatory bowel disease; OR, odds ratio; 95% CI, 95% confidence interval.
